# Tuberculosis in Sudan: a study of *Mycobacterium tuberculosis *strain genotype and susceptibility to anti-tuberculosis drugs

**DOI:** 10.1186/1471-2334-11-219

**Published:** 2011-08-16

**Authors:** Ghada S Sharaf Eldin, Imad Fadl-Elmula, Mohammed S Ali, Ahmed B Ali, Abdel Latif GA Salih, Kim Mallard, Christian Bottomley, Ruth McNerney

**Affiliations:** 1 Al Neelain University, Khartoum, P. O. Box 12702, 11121 Khartoum, Sudan; 2 Tuberculosis Reference Laboratory, TB Reference Laboratory, Ministry of Health, P. O. Box 941, 1331 Khartoum North, Sudan; 3 Khartoum Teaching Hospital, P.O. Box 102, Hospital Street, 11111 Khartoum, Sudan; 4 Department of Pathogen Molecular Biology, London School of Hygiene & Tropical Medicine, Keppel Street, London, WC1E 7HT, UK; 5 Department of Infectious Disease Epidemiology, London School of Hygiene & Tropical Medicine, Keppel Street, London, WC1E 7HT, UK

## Abstract

**Background:**

Sudan is a large country with a diverse population and history of civil conflict. Poverty levels are high with a gross national income per capita of less than two thousand dollars. The country has a high burden of tuberculosis (TB) with an estimated 50,000 incident cases during 2009, when the estimated prevalence was 209 cases per 100,000 of the population. Few studies have been undertaken on TB in Sudan and the prevalence of drug resistant disease is not known.

**Methods:**

In this study *Mycobacterium tuberculosis *isolates from 235 patients attending three treatment centers in Sudan were screened for susceptibility to isoniazid, rifampicin, ethambutol and streptomycin by the proportion method on Lowenstein Jensen media. 232 isolates were also genotyped by spoligotyping. Demographic details of patients were recorded using a structured questionnaire. Statistical analyses were conducted to examine the associations between drug resistance with risk ratios computed for a set of risk factors (gender, age, case status - new or relapse, geographic origin of the patient, spoligotype, number of people per room, marital status and type of housing).

**Results:**

Multi drug-resistant tuberculosis (MDR-TB), being resistance to at least rifampicin and isoniazid, was found in 5% (95% CI: 2,8) of new cases and 24% (95% CI: 14,34) of previously treated patients. Drug resistance was associated with previous treatment with risk ratios of 3.51 (95% CI: 2.69-4.60; p < 0.001) for resistance to any drug and 5.23 (95% CI: 2.30-11.90; p < 0.001) for MDR-TB. Resistance was also associated with the geographic region of origin of the patient, being most frequently observed in patients from the Northern region and least in the Eastern region with risk ratios of 7.43 (95%CI:3.42,16.18; p: < 0.001) and 14.09 (95%CI:1.80,110.53; p:0.026) for resistance to any drug and MDR-TB. The major genotype observed was of the Central Asia spoligotype family (CAS1_Delhi), representing 49% of the 232 isolates examined.

**Conclusions:**

We conclude that emergence of drug resistant tuberculosis has the potential to be a serious public health problem in Sudan and that strengthened tuberculosis control and improved monitoring of therapy is needed. Further surveillance is required to fully ascertain the extent of the problem.

## Background

The Republic of Sudan is the largest country on the African continent covering 2.5 million km^2 ^with a current estimated population of approximately 45 million, 42% of whom are believed to be under the age of 15 years [1]. The population is culturally and ethnically diverse with several hundred tribal groupings speaking over 130 languages [2]. The country contains 26 states divided between five geographic regions Eastern, Western, Southern, Northern and Central Sudan which includes the Capital Khartoum. However, following a recent referendum the region of South Sudan became an independent state in July 2011 [3]. During 2010 an estimated 40% of the people lived in an urbanized environment [1]. It is a poor country and gross national income per capita was less than two thousand international dollars during 2009 with a life expectancy at birth of 58 years [4]. The country has been severely affected by war, famine and flood in recent decades and has a large population of internally displaced persons [5]. It has high burden of tuberculosis (TB) with a prevalence of 209 cases per 100,000 of the population and 50,000 incident cases during 2009 [6]. The estimated adult HIV prevalence of 1.5% remains lower than that of its African neighbors to the south and a report from 2002 suggested 4% of tuberculosis patients were co-infected with HIV [5]. Tuberculosis care and treatment is provided by the National Tuberculosis Control Program under the auspices of the Ministry of Health and by a number of non-governmental organizations (NGOs) who provide care to displaced persons, including those living in refugee camps [5]. Treatment is also provided by the private sector [7]. At the time of this study the Sudan National TB Program treatment policy was for an intensive phase of rifampicin, isoniazid, pyrazinamide and streptomycin daily under direct supervision for two to three months until the patient became smear negative followed by eight months of isoniazid and ethambutol [7]. Thioacetazone was previously used in the place of ethambutol [8,9]. Patients unable to attend on a daily basis were put on a 12-month regimen excluding rifampicin. Smear-negative pulmonary patients and non severe extra-pulmonary cases were given isoniazid and ethambutol daily for twelve months, supplemented by daily streptomycin injections during the initial phase [5]. While some NGOs refer patients to the national program others provide treatment using their own regimens. TB treatment in the private sector is not regulated [7].

The emergence and spread of strains of tuberculosis that are resistant to the drugs used in standard first line treatment poses a serious threat to attempts to control the disease [10]. Drug resistance in *M. tuberculosis *arises through the selection of spontaneous mutations by inadequate therapy. Resistance to multiple drugs arises through sequential selection of mutations. Resistance to both the key drugs rifampicin and isoniazid is termed multi drug-resistant tuberculosis (MDR-TB). Patients with MDR-TB frequently fail to be cured by standard drug treatment and may remain infectious and a potential source of onward transmission. Treatment for MDR-TB requires alternative chemotherapy for at least 18 months using more expensive drugs [11]of heightened toxicity [12,13]. In Sudan treatment is provided at the Abu-Anga Teaching Hospital [14], where the drugs available are ciprofloxacin, ofloxacin, cycloserine, ethionamide and amikacin [7]. From 2005 to 2008 small numbers of patients were treated for MDR-TB [14] but in 2009 ninety four patients commenced second line treatment, thirty five were new patients and fifty nine were retreatment cases [6]. The prevalence of drug resistant TB in Sudan is not known. A previous study undertaken in central Sudan during 1965 reported high levels of resistance to the anti-tuberculosis drugs available at that time [15]. Cure rates have improved considerably since the establishment of the National Tuberculosis Control Program and introduction of short course chemotherapy, but no surveys of drug susceptibility have been reported [5,16]. Examination of isolates collected 10 years ago by molecular methods found evidence of MDR-TB in 2/39 retreatment cases [17]. The study also reported the dominance of a single *Mycobacterium tuberculosis *genotype where over half of the strains examined (29/49) shared the same spoligotype pattern.

The purpose of the current study was to investigate the susceptibly to anti-tuberculosis drugs and genotype of *M. tuberculosis *strains from patients attending three treatment centers in Sudan. Treatment history and demographic characteristics such as the geographic origin of the patient were recorded and statistical analysis undertaken to assess genotypic and demographic factors associated with the emergence of drug resistant disease.

## Methods

Scientific and ethical approval for the study was obtained from Al Neelain Ethical Review Board. Consecutive smear positive pulmonary tuberculosis patients attending out-patient chest clinics in three cities were invited to join the study. The recruitment centers were the Abu Anga Hospital (Omdurman, Central Sudan; estimated population 1.2 million), Al Shaab Teaching Hospital (Khartoum, Central Sudan; estimated population 1.9 million) and Port Sudan Hospital (Port Sudan, Eastern Sudan; estimated population 0.5 million) [18]. Study participants were enrolled three days per week, selected at random during May to December 2005. Sputum samples were requested and demographic data relating to age, gender, marital status, geographic origin of patient, tribe, occupation, housing (construction and numbers of persons) and previous history of tuberculosis was collected via a structured questionnaire. Where available HIV status was recorded but testing was not undertaken as part of this study. All participants gave their informed consent prior to submitting sputum specimens and answering the questionnaire.

The sputum specimens were decontaminated using 2% sodium hydroxide and concentrated by centrifugation prior to investigation by Ziehl-Neelsen smear microscopy [19]. For culture, sedimented samples were inoculated on Lowenstein Jensen (LJ) media containing glycerol. Following isolation and culture the *M. tuberculosis *strains obtained were screened for resistance to isoniazid, rifampicin, ethambutol and streptomycin and subjected to spoligotyping. For each patient, only the first available isolate was included in this study. Drug susceptibility testing was performed at the Sudanese National Reference Laboratory (Khartoum) for isoniazid, rifampicin, streptomycin and ethambutol by the proportion method on LJ [20]. Although the laboratory takes part in a program of external quality control the samples in this study were not sent to another laboratory for confirmatory testing.

Spoligotyping was performed on isolated cultures according to the Kamerbeek method [21] using a commercial kit to test for 43 individual spacers (Isogen, IJsselstein, The Netherlands). All DNA extracts were tested at two dilutions (1:10, 1:100) and the spoligopatterns obtained analysed using BioNumerics (Applied Maths, St-Martens-Latem, Belgium). Results obtained were compared with the international SITVIT database (Institut Pasteur, Guadeloupe). A subset of the isolates was subjected to secondary typing by MIRU/VNTR (mycobacterial-interspersed repetitive-unit variable number-tandem-repeats) [22] where variable numbers of repeats within the genome are identified following amplification by PCR. Twenty four loci MIRU/VNTR analysis [23] was performed by a commercial company, Genoscreen (Lille, France) using a high throughput DNA Analyzer (3730XL Applied Biosystems). Samples selected for secondary typing were those found resistant to rifampicin that shared a common spoligotype.

Statistical analyses were conducted in STATA version 11. Two binary outcome variables were created: 1) resistance to at least one of the TB drugs (isoniazid, rifampicin, streptomycin or ethambutol) and 2) MDR status (an individual is MDR if resistant to both isoniazid and rifampicin). Associations between these outcome variables and a set of risk factors (gender, age, case status - new or relapse, geographic origin of the patient, spoligotype, number of people per room, marital status and type of housing) were examined. To this end, risk ratios and their associated Wald-type confidence intervals were computed, and for hypothesis testing the chi-square test was used. Exact binomial 95% confidence intervals were calculated for prevalence estimates.

## Results

**Figure 1:**
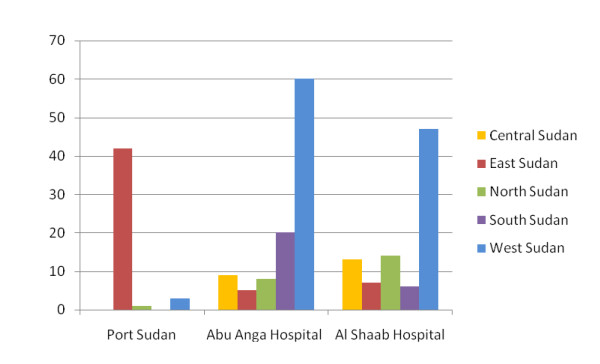
**Distribution of geographic origin of patients by recruitment centre**.

Full drug resistance data was obtained for strains from 235 patients, 232 of which were genotyped. The geographical origin of the 235 patients was unevenly spread with the Western, Eastern, Southern, Northern and Central regions providing 110 (47%), 54 (23%), 26 (11%), 23 (10%) and 22 (9%) patients respectively. The geographic spread of patients attending the recruitment centers is presented in Figure 1. Forty eight tribal groups were represented. Whereas the mix of patents attending Abu Anga and Al Shaab Hospitals was similar, the majority (83%) attending Port Sudan Hospital belonged to three pastoralist tribes not seen at the other two centers. The median age of participants was 35 years and the interquartile range was 26-45 years. One hundred and seventy five (74%) of patients were male. Reported occupations included unskilled worker or laborer 31% (n = 72), housework 21% (n = 50), business 11% (n = 26), student 10% (n = 24), unemployed 8% (n = 19), farmer 7% (n = 17), driver 5% (n = 11) or soldier 2% (n = 4). A majority of women (71%) cited their occupation as being within the home. Forty percent of males were unskilled workers or laborers and 9% were unemployed. Seventy percent (164) of the patients whose isolates were tested were classed as new patients having received less than one month of anti-tuberculosis chemotherapy. Of the remainder 51 (22%) reported having previously completed a course of TB therapy and were classed as relapsed cases, 15 (6%) had experienced interrupted treatment and 5 (2%) were treatment failures. HIV tests results were available for 27 (12%) of patients, of whom 9 (33%) were HIV positive.

### Genotyping results

DNA from 232 *M. tuberculosis *isolates were available for genotyping by spoligotyping. Results were compared with the SITVIT Database (Institut Pasteur, Guadeloupe). 160 (69%) of isolates had patterns that matched those in the database. Cluster analysis revealed that a single spoligotype pattern of the CAS1_Delhi family (SIT25) was shared by 82 individuals (35%) and that an additional 32 (14%) patients were infected with strains of the same lineage but with differing SIT numbers. The SIT25 pattern is identical to that reported previously as a being highly clustered in isolates from Khartoum [17]. Of the spoligotype patterns not found in the international database a further 12 were identified as having identical patterns to SIT25 but lacking a single spacer and for the purposes of further analysis were classified as belonging the to CAS1_Delhi lineage. The distribution of spoligotype lineages in the geographic regions of origin of the patient is presented in Table 1. The prevalence of CAS1_Delhi was highest in the Western region where it was observed in 67% of the cases examined.

**Table 1:** Regional distribution of spoligotype lineage by origin of the patient

Lineage	**Number (%) of isolates by region**.					
	
	Central	East	North	South	West	All
CAS1_Delhi*	10 (45.5)	24 (45.3)	9 (40.9)	10 (38.5)	73 (67)	126 (53.9)
T1	1 (4.5)	8 (15.1)	2 (9.1)	1 (3.9)	4 (3.7)	16 (6.9)
Beijing/W	1 (4.5)	1 (1.9)	1 (4.5)	0	4 (3.7)	7 (3.0)
H3	1 (4.5)	1 (1.9)	0	1 (3.9)	4 (3.7)	7 (3.0)
CAS	1 (4.5)	1 (1.9)	0	0	2 (1.8)	4 (1.7)
LAM7_TUR	1 (4.5)	2 (3.8)	0	0	0	3 (1.3)
U	0	3 (5.7)	0	0	0	3 (1.3)
T2	0	0	0	0	3 (2.8)	3 (1.3)
S	0	1 (1.9)	0	0	0	1 (0.4)
LAM9	0	1 (1.9)	0	0	1 (0.9)	2 (0.9)
No SIT	7 (31.8)	11 (20.8)	10 (45.5)	14 (53.9)	18 (16.51)	60 (26.3)

*Includes 114 CAS1_Delhi isolates matching SIT patterns 24, 25, 141, 232, 247, 309, 381, 1198, 1203 or 1787 and an additional 12 strains having spoligotype patterns one spacer different to SIT25.

A subset of 17 isolates found resistant to rifampicin and identified as either SIT25 (CAS1_Delhi) and 3 isolates SIT1 (Beijing/W) were subjected to secondary typing by 24 loci MIRU. The results, as previously reported elsewhere [24], demonstrated that the rifampicin resistant spoligotype families were not comprised of identical strains. The 17 isolates with identical SIT25 spoligotype patterns were subdivided into 11 MIRU types (one cluster of 5, two clusters of 2 and 8 individual MIRU types). Similarly, the 3 SIT1 isolates were found by MIRU-VNTR to be a cluster of 2 and a single strain.

### Drug susceptibility

127 patients (54%, 95% CI: 47,61) were found to have tuberculosis that was fully susceptible to isoniazid, rifampicin, ethambutol and streptomycin. The remaining 108 patients (46%, 95% CI: 39,53) had resistance to at least one of the four drugs tested while 26 (11%, 95% CI: 7,16) were classed as MDR-TB, being resistant to at least isoniazid and rifampicin. The distribution of drug resistant cases with regard to treatment status is presented in Table 2. In new cases resistance to at least one drug was observed in 43 patients (26%, 95% CI: 19,33) with 8 (5%, 95% CI: 2,8) having MDR-TB. In the 71 previously treated patients resistance to at least one drug was observed in 65 cases (92%, 95%CI: 83,101) and 17 patients (24%, 95% CI: 14,34) had MDR-TB.

**Table 2:** Susceptibility to anti-tuberculosis drugs by treatment category (Number and %)

**Treatment category**.	Pan susceptible	Resistant				MDR-TB
		
		Isoniazid	Rifampicin	Streptomycin	Ethambutol	I + R
New (N = 164)	121 (73.8)	17 (10.4)	22 (13.4)	24 (14.6)	9 (5.5)	8 (4.9)
Relapse (N = 51)	4 (7.8)	21 (41.2)	20 (39.2)	37 (72.6)	32 (62.8)	13 (25.5)
Interrupted N = 15	2 (13.3)	4 (26.7)	9 (60)	12 (80)	7 (46.7)	4 (26.7)
Failed N = 5	0	1 (20)	2 (40)	3 (60)	5 (100)	0

When compared to new cases previously treated patients had significantly increased risk of drug resistant disease, with a risk ratio of 3.51 (95% CI: 2.69-4.60) for resistance to any drug and 5.23 (95% CI: 2.30-11.90) for MDR-TB. Analysis of demographic characteristics of the population with respect to drug resistance is presented in Table 3 and for MDR-TB in Table 4. The prevalence of drug resistance was found to vary by the geographic origin of the patient, being most prevalent in patients originating from the Northern region and least prevalent among those from the East. MDR-TB was found in patients belonging to 14 of the 48 tribes represented. The Ja'alya from the Northern region had the highest number of cases (5/13; 39%) followed by the Jawama from the West (4/15; 27%). Other tribes in which MDR-TB was detected included the Dinka (2/16), Hasania (3/13), Manaseir (1/2) Masalit (1/6), Mesaria (2/10), Mima (1/1), Nawir (1/4), Nouba (1/15), Rashaida (1/4), Shaigyiah (2/15), Shamblia (1/3) and Tama (1/5).

**Table 3:** Prevalence of resistance within demographic subgroups

Variable		% RES	95% CI	RR	95% CI	P-value
SEX	Male	45.7	38.2,53.4	1		
	Female	46.7	33.7,60.0	1.02	0.74,1.40	0.898
AGE	< = 30	39.5	29.2,50.7	1		
	31-44	54.8	43.5,65.7	1.39	1.00,1.92	
	> = 45	43.1	30.8,56.0	1.09	0.74,1.60	0.118
CASE	New	26.2	19.7,33.6	1		
	Relapse	92.2	81.1,97.8	3.51	2.69,4.60	
	Failure/Interrupted	90.0	68.3,98.8	3.43	2.55,4.61	< 0.001
ORIGIN	East	11.1	4.2,22.6	1		
	West	53.6	43.9,63.2	4.83	2.23,10.47	
	North	82.6	61.2,95.0	7.43	3.42,16.18	
	South	57.7	36.9,76.6	5.19	2.28,11.83	
	Central	40.9	20.7,63.6	3.68	1.49,9.11	< 0.001
SPOLIGOTYPE	CAS1-DEL	49.1	39.6,58.7	1		
	Other	32.2	20.6,45.6	0.66	0.43,0.99	
	Unknown	53.2	40.1,66.0	1.08	0.80,1.46	0.043
MARITALSTATUS	Married	45.8	36.6,55.2	1		
	Not married	46.2	36.9,55.6	1.01	0.76,1.33	0.952
TYPEOFHOUSE	Brick	63.9	46.2,79.2	1		
	Mud	45.6	37.5,54.0	0.71	0.53,0.97	
	Shelter	36.2	22.7,51.5	0.57	0.36,0.89	0.040
PERSON/ROOM	< 6	44.4	34.9,54.3	1		
	6+	48.4	39.3,57.5	1.09	0.82,1.44	0.548

**Table 4:** Prevalence of MDR-TB within demographic subgroups

Variable		N	% MDR	95% CI	RR	95% CI	P-value
SEX	Male	175	12.0	7.6,17.8	1		
	Female	60	8.3	2.8,18.4	0.69	0.27,1.76	0.435
AGE	< = 30	86	7.0	2.6,14.6	1		
	31-44	84	17.9	10.4,27.7	2.56	1.04,6.28	
	> = 45	65	7.7	2.5,17.0	1.1	0.35,3.46	0.046
CASE	New	164	4.9	2.1,9.4	1		
	Relapse	51	25.5	14.3,39.6	5.23	2.30,11.90	
	Failure/Interrupted	20	25	8.7,49.1	5.12	1.85,14.16	< 0.001
ORIGIN	East	54	1.9	0.0,9.9	1		
	West	110	10.9	5.8,18.3	5.89	0.79,44.14	
	North	23	26.1	10.2,48.4	14.09	1.80,110.53	
	South	26	11.5	2.4,30.2	6.23	0.68,57.04	
	Central	22	18.2	5.2,40.3	9.82	1.16,82.99	0.026
SPOLIGOTYPE	CAS1-DEL	114	13.2	7.6,20.8	1		
	Other	59	8.5	2.8,18.7	0.64	0.25,1.69	
	Unknown	62	9.7	3.6,19.9	0.74	0.30,1.80	0.597
MARITALSTATUS	Married	118	9.3	4.7,16.1	1		
	Not married	117	12.8	7.4,20.3	1.38	0.66,2.87	0.393
TYPEOFHOUSE	Brick	36	13.9	4.7,29.5	1		
	Mud	149	11.4	6.8,17.6	0.82	0.32,2.08	
	Shelter	47	8.5	2.4,20.4	0.61	0.18,2.12	0.737
PERSON/ROOM	< 6	108	7.4	3.3,14.1	1		
	6+	124	14.5	8.8,22.0	1.96	0.89,4.33	0.087

Fifty three patients (23%) were found resistant to rifampicin of which 26 were also resistant to isoniazid and were classed as having MDR-TB. Thus the predictive value of rifampicin resistance for MDR-TB (i.e. the proportion of rifampicin resistant patients who had MDR-TB) was 49%. The predictive value varied by region, being 100%, 50%, 50%, 75% and 36% in the Central, South, East, North and Western regions respectively.

## Discussion

Sudan is a poor country whose healthcare delivery system has been placed under considerable strain in the past two decades through civil unrest, natural disasters and other economic factors. We present the first study to ascertain levels of drug resistant tuberculosis since the adoption of multi drug short course therapy and establishment of a DOTS program in 1992 [16]. The high levels of MDR observed in re-treatment patients (24%) and variation in the prevalence of resistance across geographic regions is a cause for concern and suggests that sub optimal treatment has been administered. Evidence of inadequate treatment is provided by a study of 19 patients attending Abu Anga Hospital during 2008 who had failed treatment with first line drugs, where over half (10) reported interrupted treatment [14]. Similarly, of 24 patients taking second line treatment for MDR-TB five had interrupted treatment once, and a further thirteen had interrupted treatment on more than one occasion. Reasons given for the interrupted treatment were lack of money, lack of drugs and feeling well. When compared to other reports from the region the prevalence of MDR in re-treatment patients is approximately double the estimate for Ethiopia to the East (12%) [25] and Kampala to the South (13%) [26] but is less that the 38% reported from Egypt, Sudan's Northern neighbour [25]. It should be noted that Northern Sudan, the region with the highest prevalence of MDR has been badly affected by both war and drought and considerable displacement of peoples has occurred. Egypt is currently host to a large Sudanese refugee and migrant population with consequent cross border movement. In the population studied those at increased risk of drug resistance included individuals of male gender and those living in a brick dwelling or sleeping in a room shared by more than six persons. Although not statistically significant these findings suggest that life style may influence the outcome of treatment and emergence of drug resistant disease.

Whilst the data from this study clearly indicates a problem with the treatment of tuberculosis in Sudan it does not provide a full picture of the situation. Three TB treatment centres were sampled. A fourth centre El Obeid Hospital in a more southern location was sampled but susceptibility tests were not completed. Sampling may have been affected by the location of the treatment centers as some rural populations may have reduced access to such urban facilities [8]. The ratio of males to female participating in the study (2.8) is higher than that reported nationally (1.7) [6] and may reflect inequalities of access to the recruitment sites. Further surveillance is needed to quantify the prevalence of resistance in Sudan and identify those areas of the country in need of strengthened TB treatment supervision. In addition, external quality assurance should be implemented for studies of susceptibility to anti-tuberculosis drugs. Testing for resistance to second line drugs was not undertaken during this study and the prevalence of XDR-TB in Sudan is not known.

Resistance to rifampicin occurs less frequently than resistance to isoniazid and in many settings may be taken as an indicator for the presence of MDR-TB [27]. The high level of rifampicin resistance in the absence of resistance to isoniazid observed in this study (11.5%) points to inappropriate therapy, where the drugs have been taken separately rather than in combination. A review of the management and supply of TB drugs in Sudan conducted in 2008 noted that appropriate treatment guidelines were often absent from treatment centres and that control of TB drugs in the private sector was lacking [7]. They also found that supply problems resulted in low stocks of combination therapy (isoniazid plus rifampicin) in some centers and that some stocks of drugs were past their expiry date. The use of rifampicin to treat other conditions such as brucellosis may also be a contributory factor [28]. The problem was most acute in patients originating from the West of Sudan which includes the regions of Kordofan and Dafur which have experienced civil unrest in recent years. It is important that expanded studies are undertaken to ascertain the prevalence of monoresistance to rifampicin in Sudan in accordance with WHO guidelines [19]. A low predictive value of resistance to rifampicin for MDR-TB in Sudan would reduce the ability to predict MDR-TB when using the new generation of molecular tests such as the Xpert MTB/RIF (Cepheid, California, USA) [29] which rely on detection of mutations in a sub unit of the RNA polymerase gene. Data from neighbouring countries is limited but in 2002 the predictive value of rifampicin resistance for MDR-TB in Egypt at 72.4% was also lower than international norms [30]. That 73% of re-treatment cases and 15% of new patients were found to have strains resistant to streptomycin suggests that the efficacy of this drug is limited in this setting. It is an injectable drug that necessitates regular attendance at a health clinic and the high levels of resistance observed suggest its role in the treatment of TB in Sudan should be reappraised.

The predominance of the CAS1_Delhi spoligotype and the number and variety of types within the lineage suggest that it has been present in the region for some time and it is likely that lineages such as Beijing/W, T and LAM7_TUR were more recently introduced. The highest proportion of CAS1_Delhi strains (66%) was observed in patients from the poorly accessible Western region but the majority of T1, LAM7_TUR and S strains were found in the Eastern region which has access to the Red Sea and international trading routes. Although not highly significant, strains belonging to the CAS1_Delhi lineage were found more likely to have developed drug resistance or MDR than those with other spoligotype patterns. It has been speculated that Beijing strains are growing in prevalence in some regions of the world and that they are prone to drug resistance [31,32]. In this study two of the seven Beijing isolates identified were found to be MDR. However, further studies shall be required to determine the significance of this observation in the Sudanese population.

Spoligotyping provided a relatively simple and rapid means of describing the population of tuberculosis strains circulating in Sudan. However, we demonstrated improved discrimination of strains sharing the CAS1_Dehli and Beijing spoligotypes when using MIRU-VNTR. For future studies we recommend that secondary typing with MIRU-VNTR be undertaken on all resistant strains having identical spoligotypes. We also observed that a batch of membranes supplied by the manufacturers consistently gave false positive results for one of the spacers (position 33). Thus although simple to perform, spoligotyping requires careful monitoring to avoid inappropriate interpretation of results.

## Conclusion

Our findings suggest that the emergence of drug resistant tuberculosis may be a serious problem in Sudan. Expanded surveillance is required to determine the prevalence of drug resistant tuberculosis in Sudan. The strong association of resistance with previous treatment observed suggests that improved monitoring of treatment and direct observation of therapy should be implemented to limit the emergence of drug resistant disease. Our findings also suggest that the origin of the patient is a risk factor for the development of resistance and surveillance should be undertaken to assess geographic differences in the effectiveness of TB treatment. The dominant *M. tuberculosis *genotype lineage in the isolates tested was CAS1_Delhi.

## Competing interests

The authors declare that they have no competing interests.

## Authors' contributions

GES participated in the conception and design of the study, performed the genotyping, undertook data analysis and interpretation of results and participated in drafting the manuscript. IF-E participated in the conception and design of the study and general supervision of the research. MA participated and assisted of the project design and sample collection. AA participated in the conception and design of the study and general supervision of the research. AS participated in assessment of the patients and data collection. KM participated in MIRU analysis and interpretation of results. CB participated in statistical analysis and drafting the manuscript. RM participated in the general supervision of the genotyping, interpretation of results and drafting the manuscript. All authors read and approved the final version of the manuscript.

## Authors' information

Dr Sharaf Eldin undertook these studies whilst working at Al Neelain University in collaboration with the Sudanese Ministry of Health National Health Laboratory. She also attended training in spoligotyping at the ZAMBART Laboratory, University Teaching Hospital, Lusaka, Zambia.

## Pre-publication history

The pre-publication history for this paper can be accessed here:

http://www.biomedcentral.com/1471-2334/11/219/prepub
